# Transactions of the Royal Academy of Medicine in Ireland

**Published:** 1889-09

**Authors:** 


*Transactions of the Royal Academy of Medicine in Ireland.* Edited by W. THOMSON, M.A., F.R.C.S. Pp. 482. Dublin: FANNIN & CO. 1888.

The handsome volume before us is one of the best of this admirable series of *Transactions*, and is altogether a credit to everyone concerned in its production. The most striking feature of the papers is their thoroughness. They are evidently prepared for a cultured audience, and bear evidence that their authors have done their work up to a high level of criticism. At least half a dozen of the articles are worthy of permanent positions, as distinctly advancing knowledge; the majority illustrate principles, impress facts, or re-kindle thought.

Where so much is good we are loth to point out defects: the defects, as might be expected, are at the hands of the most frequent contributors, and they are chiefly marked in imperfect knowledge of the work of others and a too hasty and superficial consideration of their own material. The reader will soon find out these articles for himself, and the information thus derived at first-hand will teach him to ignore articles by these men in future. The medical literary student, like every other literary student, soon finds out the men who write articles worth reading, and those who do not. It is curious to find in casual conversations what general agreement there is as to who are the medical literary bores: this knowledge cannot be acquired from reviews; it can only be got from the reading of several papers by these gentlemen. We can promise our readers an increased relative pleasure from a careful perusal of the many excellent papers in this volume, by a superficial study of the few feeble ones.

